# Reporting the Undiagnosed Cases of Hepatitis B and Hepatitis C Viruses among Patients Undergoing Elective Eye Surgery in a Specialized Eye Hospital in Egypt

**DOI:** 10.1155/2019/3985865

**Published:** 2019-07-01

**Authors:** Ahmed A. Dahab, Maha Mohamed Youssef, Hany Mohamed Eid, Khaled W. Elsadi

**Affiliations:** ^1^Cairo University, Faculty of Medicine, Cairo, Egypt; ^2^Magrabi Eye Hospital, Cairo, Egypt

## Abstract

**Introduction:**

Hepatitis B virus (HBV) and hepatitis C virus (HCV) and their long-term sequelae are considered a major health issue in Egypt. The aim of this study is to determine the prevalence of undiagnosed hepatitis B virus (HBV) and hepatitis C virus (HCV) among patients admitted for elective eye surgery in a specialized eye hospital in Cairo, Egypt.

**Materials and Methods:**

This cross-sectional study was conducted in a specialized eye hospital, Cairo, Egypt. The study included consecutive patients admitted for elective eye surgery in the period from April 2015 to June 2016. Age, sex, and procedure done were recorded for all patients. All the subjects were screened for HBV and HCV by rapid chromatography immunoassay; if positive, the results had to be confirmed by ELISA.

**Results:**

3067 patients admitted for elective eye surgery were included in the study. The mean age of the patients was 50.85 ± 19.77 years. There were 1592 (51.9%) males and 1475 (48.1%) females. The prevalence of preoperative positive HBV and HCV was 7/3067 (0.2%) and 381/3067 (12.4%), respectively.

**Conclusion:**

Given the high prevalence of HBV and HCV infection in our population in general and in this study specifically, all patients admitted for surgery should be screened for both viruses.

## 1. Introduction

Hepatitis B virus (HBV) and hepatitis C virus (HCV) and their long-term sequelae are considered a major health issue in Egypt [1].

Egypt is among the highest in the world as regards infection rate with HCV [2], where the estimated national prevalence of HCV infection among those aged 15–59 years was 7% in the most recent national survey in 2015 [3]. However, the population prevalence rate of HBV was 1.4% in 2017 [1].

Majority of infected individuals remain asymptomatic and accordingly undiagnosed leading to chronic carrier state. These carriers pose a real threat to medical personnel through self-pricks as well as to other patients sharing the same surgical instruments [4].

We conducted this study to determine the prevalence of undiagnosed HBV and HCV among patients admitted for elective eye surgery in a specialized eye hospital in Cairo, Egypt.

## 2. Materials and Methods

This cross-sectional study was conducted in Magrabi eye hospital, Cairo, Egypt. The aim of the study was to determine the prevalence of undiagnosed HBV and HCV among patients admitted for elective eye surgery. It included consecutive adult patients (≥16 years) admitted for elective eye surgery in the period from April 2015 to June 2016. Patients known to be infected with HBV and/or HCV were excluded from study. The study was approved by the hospital ethical committee and followed the declarations of Helsinki. All patients signed an informed consent before being enrolled in the study, after the nature of the study was fully explained. Age, sex, and procedure done were recorded for all patients. Thorough medical history was obtained from each patient. The data were collected from the operating room system of the Magrabi Eye Hospital.

All subjects were screened through qualitative detection of HBV surface antigen (HBsAg) and specific antibodies against HCV (anti-HCV), in the serum, using rapid chromatography immunoassay. This is a simple screening test that can be used in places with limited facilities or when rapid results are required. Results that were found positive or equivocal on screening test were confirmed by the enzyme-linked immunosorbent assay (ELISA) method for quantitative estimation of HBsAg and HCV-RNA.

### 2.1. Statistical Methods

Data were coded and entered using the statistical package SPSS (Statistical Package for the Social Science; SPSS Inc., Chicago, IL, USA) version 23. Data were summarized using mean in quantitative data and using frequency (count) and relative frequency (percentage) in categorical data [5]. For comparing categorical data, the chi-squared (*χ*^2^) test was performed.

## 3. Results

3101 patients were admitted for elective eye surgery in the period from April 2015 to June 2016. Thirty-four patients were excluded due to infection with either HCV or HBV. 3067 patients were included in the study. The mean age of the patients was 50.85 ± 19.77 years. There were 1592 (51.9%) males and 1475 (48.1%) females. The prevalence of preoperative positive HBV and HCV was 7/3067 (0.2%) and 381/3067 (12.4%), respectively. No statistically significant difference was found as regards the prevalence of HBV and HCV among males and females, *p* value 0.216 and 0.059, respectively, as shown in Table 1.

Patients were further stratified into 3 groups according to age: Group 1: 16–29 years; Group 2: 30–60; and Group 3: above 60 years. The HBV- and HCV-positive patients were 0% (0/486) and 0.6% (3/486), 0.3% (5/1475) and 13.8% (204/1475), and 0.2% (2/1106) and 15.7% (174/1106) in 3 groups, respectively. The difference in the prevalence of HBV among different age groups did not reach statistical significance (*p* value 0.365); however, it was statistically significant for HCV (*p* value < 0.001).

The patients underwent eye surgeries which included 1660 (54.12%) anterior segments, 1017 (33.16%) posterior segments, and 390 (12.72%) oculoplastic and strabismus surgeries. The HBV- and HCV-positive patients were 2 (0.12%) and 218 (13.1%), 3 (0.29%) and 149 (14.7%), and 2 (0.5%) and 14 (3.6%), respectively.

## 4. Discussion

Egypt is facing a huge burden of HBV and HCV infections [1, 3]. Despite this tremendous burden, majority of patients with chronic HCV and HBV are undiagnosed and asymptomatic [1, 6]. This study was conducted to determine the prevalence of undiagnosed HBV and HCV among patients admitted for elective eye surgery in a hospital in Cairo, Egypt. The prevalence of preoperative positive HBV and HCV was found to be 0.2% and 12.4%. These prevalence rates of HBV and HCV are different from those released by the most recent national surveys, being 1.4% and 7% [1, 3], respectively. HBV and HCV prevalence rates varied considerably according to age, gender, and geographical location. Infection risk tends to increase with age [1, 3]. Population prevalence was estimated among adults aging up to 60 years; however, our study population included many older patients due to the nature of eye diseases that increase in elderly, which might have led to discrepancy in estimated prevalence. Moreover, the current study was conducted in Cairo which might have different prevalence rates due to demographic factors. There is generally higher prevalence of HBV in Upper Egypt where prevalence rates are highest in Aswan and Asyut. This is in contrast to HCV for which higher prevalence was found in Lower Egypt, especially in Menoufia and Damietta [1, 3]. It is worth noting that we have excluded those known to be infected before surgery.

As for HBV, more females were infected when compared to males; however, the sample size of HBV infected patients was too small (7 patients) to yield significant results. On the contrary, the prevalence rate for HCV among males is higher, compared to that for females, even though this did not reach statistical significance. Previous studies showed that more males are affected in comparison to females, as regards the national prevalence rate for both HBV and HCV, in Egypt [1, 7]. Similar results have been suggested in other endemic countries such as Pakistan [8–11].

In this study, the highest prevalence of HBV was found in the age group ranging from 30 to 60 years, while those above 60 years were having the highest prevalence of HCV. In Egypt, infection risk tends to increase with age, to a peak among those aged 35–44 and 55–59, for HBV and HCV, respectively [1, 3]. Similar studies, in Pakistan, have shown that the highest prevalence of HBV and HCV is found in the age groups, 40–60 [8], 50–85 [9], and 30–60 [12]. We have not included patients below 16 years, in the study, due to low prevalence of infection among them due to the various health interventions implemented by the Ministry of Health since 2008 [3] and high compulsory HBV vaccination coverage [13].

HBV and HCV are blood-borne viruses that can be transmitted through transfusion of blood, sharing needles for intravenous drug use, hemodialysis, and sexual activity [14]. However, it is not common for HCV to be transmitted sexually. Moreover, HBV and HCV are a well-reported occupational hazard for medical personnel. In health care settings, transmission of HBV and HCV has been documented from patient to medical personnel, from medical personnel to patient, and from patient to patient [8]. Medical personnel are highly exposed to infection risk due to injuries from contaminated sharp surgical instruments, such as needles or blades [15, 16].

In ophthalmic surgeries, patient-to-patient infection can occur through disposable phacoemulsification accessories, which can be a vector of transmission of infectious diseases caused by various organisms, especially with ignoring exchange of parts of accessories in between surgeries [17]. An association between HCV infection and eye surgeries, especially cataract removal, has been reported [18]. Moreover, HBV infection after corneal transplantation has been documented as well [19].

Risk factors that may be related to HBV and HCV infection, such as previous blood transfusion, drug use, and sexual behavior, have not been investigated which is considered a limitation of this study. The strength of this study is being the first one, to the best of our knowledge, to address this issue in patients undergoing elective eye surgeries in our community.

## 5. Conclusion

Given the high prevalence of HBV and HCV infection in our population in general and in this study specifically, all patients admitted for surgery should be screened for both viruses. Moreover, the majority of infected individuals are undiagnosed and can spread infection to medical personnel and other patients in health care setting, so efforts are to be made for adequate training of medical personnel concerning the risk of infection, and the necessary precautions should be strictly applied to avoid the spread of infection. Sterilization of medical instruments and careful use of disposable surgical equipment should be efficiently implemented, especially in developing countries where there is high turnover of patients.

## Figures and Tables

**Table 1:** Prevalence of HBV^1^ and HCV^2^ among males and females undergoing elective eye surgery.

	HCV		HBV	
	Negative (%)	Positive (%)	Negative (%)	Positive (%)
Male	86.49	13.51	99.87	0.13
Female	88.75	11.25	99.66	0.34

^1^Hepatitis B virus; ^2^hepatitis C virus.

## Data Availability

All data and supplementary data are available from the corresponding author upon request.
